# Integrating environmental sustainability into health technology assessment: an international survey of HTA stakeholders

**DOI:** 10.1017/S0266462324000631

**Published:** 2024-11-29

**Authors:** Michela Bobini, Americo Cicchetti

**Affiliations:** Graduate School of Health Economics and Management (ALTEMS), Università Cattolica del Sacro Cuore, Rome, Italy

**Keywords:** technology assessment, environment, healthcare evaluation methods, survey, sustainability

## Abstract

**Introduction:**

Health technologies play a relevant role in environmental sustainability (ES). However, limited evidence exists on approaches and methods to integrate ES into the Health Technology Assessment (HTA).

**Objectives:**

The purpose of this study is: (i) to provide an overview of global HTA organizations’ progression toward the integration of ES into HTA; (ii) to investigate various paths for this integration, highlighting obstacles, priorities, potential approaches, and methods.

**Methods:**

Data were collected via questionnaires from organizations belonging to HTA networks, International Network of Agencies for Health Technology Assessment, and European Network for HTA. To complement the results of the survey, the authors carried out a desk analysis with strategic documents available on institutional websites.

**Results:**

The survey included twenty-six respondents from twenty different countries (thirty-three percent response rate). Among the study’s participants, there is a notable acknowledgment of the importance of integrating ES into HTA. However, only nine organizations are actively engaged in these integration efforts, each employing unique methodologies and perspectives. There is a substantial consensus on the application of life cycle assessment, with a particular emphasis on the use of environmentally extended input–output analysis, and a stronger preference for cost-utility analysis. Nevertheless, evidence on integrating ES into HTA remains scarce. Major challenges identified include data collection difficulties and the necessity for interdisciplinary teams.

**Conclusions:**

Our study represents a preliminary effort to systematize initiatives aimed at integrating ES into HTA. Further research is required to customize methods and tools for appropriately evaluating the environmental impacts of technologies. The findings suggest that achieving ES-HTA integration demands a multi-tiered, interdisciplinary approach.

## Introduction

The correlation between the environment and health outcomes has long been documented in epidemiologic literature (1–10). The World Health Organization (WHO) (11) reported that “Climate change is already impacting health in a myriad of ways, including death and illness from increasingly frequent extreme weather events, such as heatwaves, storms, and floods, the disruption of food systems, increases in zoonoses and food, water and vector-borne diseases, and mental health issues.” WHO (12) estimates that “environmental impacts are expected to cause approximately 250,000 additional deaths per year, between 2030 and 2050”. In the last decades, the most recent agreements have shown that environmental sustainability (ES) has become the common pivot of the international economic and political agenda (13–16). Likewise, healthcare policymakers have also begun addressing ES. The National Health Service (NHS) in England has established ambitious net zero targets: by 2040 for directly controlled emissions and by 2045 for those with limited influence (17). The NHS has already reduced its carbon footprint by sixty-two percent from 1990 levels through a combination of top-down and bottom-up initiatives, which continue to evolve to this day (17–19). Meanwhile, the Dutch Green Deal aims to minimize waste and emissions within healthcare settings and the Swedish government is planning the use of more environmentally friendly pharmaceuticals through an eco-classification system (20;21). The healthcare sector is becoming increasingly environmentally conscious, even in countries lacking explicit ES policies: some private life science companies are taking spontaneous initiatives to become “greener,” while some countries like Germany are adopting bottom-up approaches with individual climate champions leading sustainable healthcare initiatives (22–24). The healthcare sector extensively utilizes Health Technology Assessment (HTA) to ascertain that its economic investments in novel pharmaceuticals, medical devices, or care models, are appropriate in relation to patients’ anticipated health benefits (25). The integration of HTA into healthcare decision-making processes not only enhances the evaluation of healthcare technologies but also promotes sustainability, equity, and comprehensive resource allocation (26;27). Expanding the set of factors considered in HTA would therefore jointly maximize health outcomes and social welfare, ultimately improving the overall value proposition of healthcare systems (25;28;29). Consequently, “there is increasing interest among HTA agencies to more frequently or consistently incorporate environmental considerations into assessments of health technologies” as highlighted by Dufour, Weeks, De Angelis, Marchand, Kaunelis, Severn et al. (30). In the literature, carbon dioxide (CO2) emissions are the most frequently discussed environmental aspect, constituting about seventy-five percent of all global greenhouse gases (GHGs). Consequently, CO2 is commonly used as a unit of measurement for CO2 equivalent (CO2e) (31–34). CO2e allows for the comparison of emissions from various GHGs based on their global warming potential (GWP). It converts the amounts of different gases to the equivalent amount of CO2 with the same GWP, as specified by the International Panel on Climate Change (35;36). Estimates of CO2e may pertain to healthcare institutions and their associated supply chains or may focus on specific health services or interventions (25;37–44). However, evidence about approaches and methods to integrate ES into HTA remains sparse (8;45–48). The comprehensive perspective provided by life cycle analysis (LCA) prevails in academic debate, at least at the theoretical level (25;29;34;46;48;49). The method evaluates environmental effects across all stages of the life cycle of a technology, encompassing raw material extraction, manufacturing, distribution, utilization, and potential recycling or disposal. An LCA incorporates a broad range of environmental categories, including GHGs emissions, particulate matter, ozone depletion, human toxicity, ecotoxicity, acidification, and eutrophication (25;50). For instance, Marsh, Ganz, Nørtoft, Lund, and Graff-Zivin (29) suggest the application of LCA to measure ES. Specifically, they propose employing environmentally extended input–output analysis (EEIOA) to estimate carbon emissions per unit of output within an industry, or alternatively, utilizing a process analysis technique that entails a thorough examination of environmental impacts across the entire life cycle, including considerations such as raw material utilization and energy consumption. However, assessments are more frequently limited to carbon footprint evaluations, which encompass the same life cycle stages of a health technology but only account for CO2e emissions, excluding other environmental impacts. McAlister, Morton, and Barratt (25) illustrate two methods of undertaking LCA: EEIOA which relies on country-specific economic input/output tables to estimate the average carbon emissions associated with goods or services exchanged within a particular sector, and process-based LCA (P-LCA) that estimates carbon emissions at a detailed level for each activity carried out within interconnected processes throughout the value chain. Additionally, other studies have emphasized the possibility of adopting a hybrid methodology that combines input–output-based analysis (using macroeconomic analysis) and process-based analysis (using specific carbon emission attributes throughout the product’s life cycle) to overcome challenges in environmental data acquisition (51;52). Assessing the environmental impact of health technologies requires the identification of outcome measures that, in addition to clinical utility, effectiveness, efficiency, or satisfaction, also quantify the environmental impact of medical interventions (i.e., green metrics) (28). Different methods and deliberative processes emerge from the literature, appropriately adapted for this purpose: (a) Cost-utility analysis (CUA) (29;37;46;48;53), which estimates the marginal health gains associated with the marginal environmental improvements of using one technology rather than another; (b) Cost–benefit analysis (CBA) (29;37;46;48), which converts all outcomes into monetary units, thereby capturing and allowing direct comparison between a wide range of social costs and benefits; (c) Multicriteria decision analysis (MCDA) (25;29;37;46;48), which encompasses a range of different methods, including, among others, the analytic hierarchy process, the analytic network process, the multi-attribute utility theory, the multi-attribute value theory, outranking, the social multicriteria evaluation, and the technique for order of preference by similarity to ideal solution. Uncertainty extends beyond methodology choice, to include the number of HTA organizations involved in ES inclusion, their approaches, partnerships, and current progress. In this context, the purpose of this research is twofold: (i) to provide an overview of global HTA organizations’ progression about ES integration into HTA; (ii) to explore the different trajectories that would allow the integration of ES into HTA, by identifying obstacles, priorities, possible approaches, and methods.

## Methods

To achieve the aforementioned objectives, a semi-structured survey was administered to HTA organizations. This questionnaire was presented and discussed at two different academic conferences held in September and October 2022 (XXVI conference—AIES Italian Association of Health Economics and XV conference SIHTA—Italian Society of HTA). To complement the results of the survey, the authors also carried out a desk analysis of the strategic documents available on the institutional websites of the participating HTA organizations.

### Selection of respondents

Organizations were selected based on their membership to the International Network of Agencies for HTA (INAHTA) and European Network for HTA (EUnetHTA), as of October 2022. For dissemination, the survey was published in the INAHTA newsletter in October 2022. In addition, in November 2022, April 2023 and September 2023, it was also sent to organisations’ e-mails retrieved from their websites. Organizations without e-mail addresses were therefore excluded. The survey was received by seventy-nine organizations, including agencies, research centers, government departments, and other institutions involved in HTA. The link to the survey remained active from October 2022 to October 2023. This period was necessary to ensure adequate participation from a diverse range of experts in HTA across various national organizations.

### Structure of the questionnaire

The survey was developed according to specific requirements of questionnaire formatting (54–56). It contained eighteen questions, of which thirteen were multiple-choice and five open ones (see Supplementary Material). It was divided into two sections and built with the qualtrics.com platform. The time for completion was approximately 25 minutes. After gathering demographic information and inquiring whether organizational behavior in relation to the topic was influenced by any pertinent governmental mandates, Part I assessed the maturity level of the ES-HTA integration for each organization. This section was developed according to the theories of implementation science (57–59). The study adapted Rogers’ Diffusion of Innovations theory (58), to consider four stages which an organization faces, when deciding whether to adopt an innovation (in this case the innovation is integrating ES into HTA): (i) *knowledge*—the organization becomes aware about the importance of integrating ES in HTA; (ii) *persuasion*—the organization forms an opinion and takes a position about the integration of ES into HTA; (ii) *decision*—the organization takes action to pursue the integration of ES into HTA (e.g., formalization of procedures in strategic documents); (iv) *implementation—*the organization has somehow worked to integrate ES into HTA. This theory has been extensively applied to understand knowledge transfer in clinical practice. Recently, its scope has also expanded to explain the adoption of evidence in healthcare settings and policy formulation (60–62). Applying the Rogers framework to integrate ES into HTA offers a structured approach to better identify determining factors. The last question about implementation functioned as a filter: those answering “no” finished the questionnaire, whereas those answering “yes” were invited to describe the activities undertaken in favor of the ES-HTA integration, the areas of expertise of involved actors, and any organizational changes made. Part II of the survey explored trajectories for the ES-HTA integration, seeking obstacles, priorities, and approaches. Specifically, respondents were first asked to rank barriers and hindering factors according to importance; then, they were asked to select for which assessable technologies, it was more important to insert the ES dimension. Then, respondents were asked to express the likelihood of them adopting alternative methods and approaches for the integration. These questions are Likert-type items based on validated scales from “very unlikely” to “very likely”. Authors selected the most common approaches and evaluation methodologies to integrate ES into HTA, from those emerging in the literature (29;46;48;53). The predominant method to assess the environmental impacts of health technologies is LCA, which therefore absorbs the focus of this study in two forms: EEIOA and P-LCA (25;29). Furthermore, the study also considers CUA, CBA, and MCDA (25;29;37;46;48), which are described in Table 1 (63–65). The final questionnaire question was left open for further comments.

**Table 1.** Description of different approaches considered in the methodology

Methods	Description
Cost–Benefit Analysis (CBA)	CBA evaluates healthcare interventions by comparing the total costs with the total benefits, both expressed in monetary terms. This method allows for the assessment of the economic value of different interventions, enabling decision-makers to allocate resources to those with the highest net benefits. CBA is advantageous for its ability to incorporate a wide range of outcomes into a single monetary metric, facilitating clear comparisons. However, it can be challenging to assign monetary values to health outcomes and other non-market effects.
Cost-Utility Analysis (CUA)	CUA assesses the cost and outcomes of healthcare interventions using quality-adjusted life years (QALYs) as the unit of measurement. This approach standardizes outcomes, allowing for the comparison of different interventions based on their ability to improve both the quantity and quality of life. Despite its utility, the method relies on the accurate measurement of QALYs, which can be subjective and influenced by individual patient preferences.
Multi-Criteria Decision Analysis (MCDA)	MCDA is a comprehensive approach that evaluates and prioritizes healthcare interventions by incorporating multiple criteria. This method integrates both quantitative and qualitative factors, allowing for a holistic assessment that considers diverse stakeholder values and preferences. MCDA facilitates transparent and structured decision-making processes, accommodating complex and multi-dimensional healthcare decisions. While it is highly versatile, the effectiveness of MCDA depends on the careful selection and weighting of criteria, which can introduce subjectivity and require robust stakeholder engagement.

*Abbreviations:* CUA, cost-utility analysis; MCDA, multi-criteria decision analysis; CBA, cost–benefit analysis.

## Results

The survey was completed by twenty-six respondents from twenty countries: Argentina (*n* = 1), Australia (*n* = 2), Belgium (*n* = 1), Canada (*n* = 2), England (*n* = 2), Finland (*n* = 1), France (*n* = 1), Germany (*n* = 1), Hungary (*n* = 1), Italy (*n* = 1), Japan (*n* = 1), Lithuania (*n* = 1), Malaysia (*n* = 1), Malta (*n* = 1), Netherland (*n* = 1), Poland (*n* = 1), Spain (*n* = 2), Sweden (*n* = 1), Turkey (*n* = 3), and USA (*n* = 1). The response rate was thirty-three percent. As shown in Table 2, respondents included a variety of organizations active or involved in HTA: agencies, research centers, institutes, and governmental departments. The roles of respondents in each organization were heterogeneous: eighteen respondents were directors, three had a specific role in ES-HTA integration and five held other roles such as principal scientist, senior planning officer, and researcher.

**Table 2.** Survey participants (organization name and country)

HTA organizations	Country
The Institute for Clinical Effectiveness and Health Policy (IECS)	Argentina
Adelaide Health Technology Assessment (AHTA)	Australia
Australian Safety and Efficacy Register of New Interventional Procedures – Surgical (ASERNIP-S)	Australia
Belgian Health Care Knowledge Centre (KCE)	Belgium
Canadian Agency for Drugs and Technologies in Health (CADTH)	Canada
Institut National d’Excellence en Santé et en Services Sociaux (INESS)	Canada
Finnish Coordinating Center for Health Technology Assessment (FINCCHTA)	Finland
Haute Autorité de Santé (HAS)	France
The Federal Joint Committee (Gemeinsamer Bundesausschuss) (G-Ba)	Germany
National Insitute of Pharmacy and Nutrition (Department of Health Technology Assessment)	Hungary
Agenzia Italiana del Farmaco (AIFA)	Italy
Center for Outcomes Research and Economic Evaluation for Health (C2H)	Japan
Institute of Hygiene	Lithuania
Health Technology Assessment Section, Ministry of Health (MAHTAS)	Malasyia
Directorate for Pharmaceutical Affairs, Ministry for Health Malta	Malta
The National Health Care Institute (ZIN)	Netherland
Health Policy Institute	Poland
Agència de Qualitat i Avaluació Sanitàries de Catalunya (AQUAS)	Spain
Basque Office for HTA (OSTEBA)	Spain
Swedish Agency for Health Technology Assessment and Assessment of Social Services (SBU)	Sweden
Turkish Ministry of Health Technology Assessment Department	Turkey
Izmir Katip Celebi University Health Applied and Research Center	Turkey
Turkish Evidence Based Medicine	Turkey
National Institute for Health and Care Excellence (NICE)	England
National Institute for Health Research (NIHR)	England
Agency For Healthcare Research and Quality (AHRQ)	USA

### HTA organizations’ progression toward an ES-HTA integration

Table 3 illustrates HTA organizations’ progression towards ES’s integration into HTA, according to Rogers’ framework of innovation decision process. Regarding the knowledge and awareness about the ES topic, the majority of respondents (sixty-nine percent) were in favor of integrating ES into HTA. The rest (thirty-one percent) could “maybe” be so, whereas no one expressed opposition. Regarding the positions taken by the organizations, only eleven were somehow effectively considering such integration, six may consider doing so in the future, and nine will not at all. Among the eleven organizations in favor, only one received top-down instructions from the national government. The survey investigates the reasons why organizations might consider the integration, giving three options: (i) because environmental changes could directly affect people’s health; (ii) because policy-makers have broad mandates and objectives extending beyond health care; (iii) all of the above. The majority (fifty-eight percent) of respondents selected “all of the above”. Two organizations selected either people’s health or policy-maker mandates as their rationale.

**Table 3.** Summary of different stage of innovation-decision process the organizations are about integrating ES into HTA

Stage of innovation decision process	Survey’s questions	Answers		
		Yes	Maybe	No
Knowledge	Are you in favor of incorporating ES into HTA?	18 (69%)	8 (31%)	0
Persuasion	Is your organization somehow considering to include the ES dimension into HTA?	11 (42%)	6 (23%)	9 (35%)
Decision	Has the integration of ES into HTA been formally included in your organization’s strategic objectives?	6 (23%)	–	20 (77%)
Adoption	Has your organization worked (or is currently working) on integrating ES into HTA?	9 (35%)	–	17 (65%)

*Note:* this part of the survey was completed by all 26 HTA organizations. The table shows the distribution of answers for each question. The answers are closed: “yes”, “maybe,” and “no”. Both absolute values and percentages of the total are shown.

The survey considers the formalization of the objective within the strategic documents of organizations as a surrogate indicator for the decision to integrate ES into HTA. Only six organizations (twenty-three percent) met this criterion. The formalization of these objectives is delineated herein, enhancing the survey findings through supplementary desk analysis of organizations’ strategic documents. For instance, within the School of Population Health at Adelaide University, AHTA’s strategic plan outlines the incorporation of ES as an area of focused study in its research domains, including HTA (66). CADTH underscores the importance of anticipating decision-maker’s needs to understand available evidence, identify gaps, and address challenges for the implementation of optimal solutions (67). To achieve this, CADTH intends to include diverse perspectives such as equity, environmental, and patient considerations, in line with its strategic objectives. The organization also commits to staying attuned to evolving social values, acknowledging the impact of social determinants on health outcomes, and the environmental footprint of health systems, and exploring avenues to mitigate existing or potential inequities through technology implementation. NICE highlights the necessity of incorporating ES into their assessments and recommendations, alongside health economic considerations (68). Specifically, NICE expresses readiness to integrate ES and societal values into their guidance, explore novel approaches to understand and utilize patient and public opinions, and globally take a leading role in adopting environmental impact data to mitigate the carbon footprint of health and care services. ZIN advocates for efficient resource utilization, including personnel, materials, and finances, as a core principle of effective healthcare delivery to ensure system sustainability (69). HAS declares its commitment to promoting an enhanced consideration of environmental concerns across its various initiatives (70). Specifically, HAS endeavors to furnish healthcare professionals with improved decision-making instruments and advocates for redefining the criteria for quality and safety of care and support, as well as for necessary adaptations within the health system to address ES considerations. OSTEBA affirms the formalization of integrating ES into HTA as a strategic objective. However, no documentation regarding this integration is presently accessible on the organization’s website.

The survey reveals that nine organizations, namely AHRQ, AHTA, ZIN, OSTEBA, NIHR, NICE, HAS, CADTH, and the Poland Health Policy Institute, have either undertaken or are currently engaged in efforts to integrate ES into HTA. Of these, five organizations (AHRQ, AHTA, ZIN, OSTEBA, and Poland Health Policy Institute) are reviewing evidence pertaining to ES integration. Having developed guidelines over a decade ago, NIHR has demonstrated a longstanding commitment to carbon reduction, also initiating a program to encourage researchers to address ES in their projects. Similarly, NICE has pursued various research projects focused on identifying relevant domains (environmental outcomes) and quantification techniques for HTA analysis and decision-making, understanding public perceptions of ES integration, and refining organizational strategies. HAS has published methodological guidelines delineating how to identify and classify organizational impacts, including environmental ones. CADTH is actively developing an approach for ES-HTA integration by assessing key environmental considerations that could significantly influence health technology decisions.

### Organizational changes and competences to integrate ES into HTA

Henceforth, the results of the survey refer exclusively to the nine aforementioned organizations working on ES. The survey examines organizational actions for an ES-HTA integration. Six organizations assigned the task to existing teams. ZIN formed a dedicated team through the reorganization of existing units, while NICE strengthened an existing unit through new hires. Lastly, NIHR recruited new staff to build a new team. Such teams encompass multidisciplinary experts, including doctors, pharmacists, economists, and engineers. Other appreciated competencies embrace past experiences in health policy, public heath, HTA, research methodology, data science, patient engagement, information science, and leadership.

### Priority application area for HTA integrated with ES

For each assessable technology (according to HTAglossary.net, the list comprises: devices, medicines, vaccines, procedures, programs, and systems), respondents were asked to express the importance of inserting the ES in the HTA process. The vote was expressed on a scale from 1 to 7, where 1 = item where it is a higher priority and 7 = item where it is of least priority.

Considering average scores, the areas are prioritized in this order: device (2.4), procedure (2.8), medicine (3.3), system (3.4), program (4.0), and vaccine (5.0).

### Likelihood of adopting different approaches and methods for ES-HTA integration

Participants widely agreed on using LCA to integrate ES into HTA (on a scale of 1 to 10, the likelihood of adoption was 7.5 on average). Table 4 reports average “likelihood scores” to adopt EEIOA rather than P-LCA, to undertake an LCA. The table also shows average ‘likelihood scores’ for adopting CUA, MCDA, or CBA for the ES-HTA integration. EEIOA emerges as the preferred strategy to evaluate environmental impacts throughout the life cycle of a technology, scoring 6.0 on average, while process analysis scored 4.7. Additionally, the CUA is the preferred methodology to measure ES impacts, scoring 6.1. MCDA follows with 5.1 and, lastly, CBA scored 4.8. However, there is considerable variability among the participants’ responses.

**Table 4.** Likelihood to adopt different approaches and methods for an ES-HTA integration

Approaches and methods		Av.	Min.	Max.	SD
Approaches	EEIOA	6.0	5.0	8.0	1.2
	P-LCA	4.7	3.0	8.0	1.9
Methods	CUA	6.1	3.0	9.0	2.2
	MCDA	5.1	1.0	9.0	2.6
	CBA	4.8	2.0	9.0	2.3

*Note:* The table displays the results of the survey completed by the nine HTA organizations that are working on integrating ES into HTA. Respondents were asked to express their likelihood to adopt each strategy (approaches and methods) on a scale where 1 = very unlikely and 10 = very likely.

*Abbreviations:* Av., Average score of respondents; Min., minimum score assigned by respondent; SD, standard deviation of the scores; EEIOA, environmentally extended input–output analysis; P-LCA, process analysis across the life cycle; CUA, cost-utility analysis; MCDA, multi-criteria decision analysis; CBA, cost–benefit analysis.

### Importance of hindering factors in ES-HTA integration

All the inhibiting factors (53) enumerated in the survey were given high importance, as evidenced by their respective ratings in Table 5. According to the results, we can reconstruct the following relevance ranking of hindering factors: (i) scarce availability of data or difficulty in tracing data back to a specific technology; (ii) environmental data collection can be labor intensive and time-consuming; (iii) unfamiliarity with the approaches available; (iv) absence of scientific consensus on the most appropriate integrative approach to capture environmental impacts of technology; (v) lack of awareness about the relevance of integrating ES into HTA.

**Table 5.** Importance of hindering factors to integrate ES into HTA

Hindering factors	Av.	Min.	Max.	SD
Scarce availability of data or difficulty in tracing data back to a specific technology	9.0	8.0	10.0	0.9
Environmental data collection can be labor intensive and time-consuming	8.3	7.0	9.0	0.7
Unfamiliarity with the approaches available	8.1	5.0	10.0	1.7
Absence of scientific consensus on the most appropriate integrative approach to capture environmental impacts of a technology	8.0	6.0	10.0	1.3
Lack of awareness about the relevance of including ES into HTA	7.3	4.0	10.0	1.7

*Note:* the table displays the results of the survey completed by the nine HTA organizations that are working on integrating ES into HTA. Respondents were asked to evaluate the importance of each factor according to a scale where 1 = not important at all ad 10 = very important.

*Abbreviations*: HTA, Health Technology Assessment; Av., Average score of respondents; Min., minimum score assigned by respondent; SD, standard deviation of the scores.

### ES into HTA models

HTA models are often structured according to different domains and then articulated into topics. For example, in the EUnetHTA’s HTA Core model (71), ES is only hinted at in two topics of the security domain “C0040—what kind of risks for public and environment may occur when using the technology?” and “C0064—How can one reduce safety risks for the environment?”. Therefore, four respondents believe it would be more appropriate to integrate ES transversally to the different domains by creating topics, while five chose to develop a separate domain dedicated to ES.

## Discussion

### Main findings

The application of HTA to support healthcare decision-making processes enhances the evaluation of healthcare technologies, thereby promoting sustainability, equity, and efficient resource allocation (26;27). Integrating ES into HTA constitutes a progressive step toward enhancing health outcomes and social welfare, thereby augmenting the overall value of healthcare systems (25;53). The study aimed to offer a comprehensive understanding of how global HTA organizations are advancing the integration of ES into HTA. Additionally, we sought to investigate various paths for integrating ES into HTA, highlighting obstacles, priorities, potential approaches, and methods. The overall response rate for the survey was thirty-three percent, encompassing a total of 26 HTA organizations. This relatively low response rate may indicate varying levels of engagement or interest in ES across the broader HTA community. Nevertheless, among the participants, the findings highlight a widespread acknowledgment of the significance of integrating ES into HTA. As Marsh, Ganz, Nørtoft, Lund, and Graff-Zivin (29) assume, this recognition stems from the perception that environmental shifts can directly impact human health on one side, and, on the other, from the fact that policymakers’ expansive mandates and objectives extend beyond the borders of healthcare provision. Notably, all respondents manifest a favorable stance, or at least a tentative interest, in incorporating ES considerations into HTA processes. Despite the pervasive awareness of this imperative, the study elucidates a considerable gap between awareness and active engagement among participants. Specifically, seventeen organizations (sixty-five percent of respondents), are presently contemplating some form of integration of ES within HTA frameworks. Furthermore, the practical implementation of such integration remains markedly limited, with nine organizations actively embedding ES considerations into HTA practices. The prevailing trend is in line with the most recent research in this field (25;28;29;44;48), and involves learning about the body of evidence on ES applied to HTA, to refine existing approaches and strategies. Notably, the frontrunners are NIHR and the NICE, exhibiting a heightened level of advancement in incorporating ES considerations into their operational frameworks. This advancement is bolstered by governmental support toward longstanding commitments to achieving net zero emissions (17). Additionally, the CADTH is actively engaged in formulating an approach for the integration (67). However, it is worth noting that, as evidence regarding approaches and methods remain scant (25;29;46;48), most organizations are still only familiarizing with the subject matter, with ongoing processes of understanding and studying the intersection of ES and HTA. The survey results revealed preferences discerned from the academic discourse, surrounding approaches, and methodologies aimed at integrating ES into HTA. Specifically, there is substantial agreement on the utilization of LCA (25;29), with a particular emphasis on the adoption of EEIOA. Meanwhile, concerning ES impact evaluation, our survey participants demonstrated a greater inclination towards CUA, MCDA, and CBA (25;29;37;46;48), in that order. Nevertheless, determining an optimal approach remains premature. This is further compounded by the recognition that methodology selection may vary depending on the specific technology being assessed, requiring additional consideration of the feasibility of various methods. Consistent with what Marsh, Ganz, Nørtoft, Lund, and Graff-Zivin illustrated (29), among the most pertinent obstacles in this integration are the constrained availability of data or challenges associated with tracing data to specific technologies: data collection poses significant challenges due to its labor-intensive and time-consuming nature. Addressing these obstacles is paramount for advancing the integration of ES considerations into HTA, thereby facilitating informed decision-making within healthcare contexts. Moreover, HTA organizations might require enhancing their current teams with supplementary competencies (e.g., climate engineers). Consistently with what other authors have already argued (25;29;48), it is fundamental that personnel within HTA teams exhibit diverse backgrounds, reflecting the interdisciplinary essence inherent in addressing sustainability considerations within healthcare settings.

### Strengths and limitations

The main contribution of this study is its capacity to offer a comprehensive overview of the progress made by HTA organizations in integrating ES into HTA. This facilitates opportunities for knowledge exchange and learning from best practices, while also shedding light on the ongoing discourse surrounding methodological approaches and the challenges that need to be addressed. However, several limitations warrant consideration. Firstly, the overall respondent rate for the survey was thirty-three percent. This relatively low response rate may introduce potential biases and limit the generalizability of the findings. Specifically, it might reflect a lack of engagement or differing levels of interest in the subject matter across the wider HTA community, which could affect the representativeness of the results. Consequently, the insights drawn from the survey should be interpreted with caution, acknowledging the potential for non-response bias. Nonetheless, it is commendable that representation from twenty different countries is achieved. Additionally, it is essential to acknowledge that HTA is a multifaceted discipline, operating at various levels. Although the study focuses on the national level, insights from the hospital-based HTA could offer valuable perspectives. These insights have the potential to promote cross-sectoral solutions that integrate HTA with environmental management strategies. Finally, it is crucial to recognize that the field of study is rapidly evolving, and advancements may have occurred subsequent to the survey period.

## Conclusions

Health technologies play a relevant role in ES. Our study serves as an initial step in systematizing the endeavors aimed at integrating ES into HTA. The findings indicate a limited engagement or varying levels of interest in ES within the broader HTA community. However, among the participants of the study, there is notable recognition of the importance of such integration. Only nine organizations are actively engaged in these efforts, and each adopts distinct approaches and perspectives. Although current methods and tools have already proven their potential, additional research is essential for their customization, and for appropriate evaluations of the environmental and human health effects of technologies. In this context, encouraging cross-border discourse, facilitated by international HTA networks, stands as a potential pivotal mechanism for fostering scientific consensus towards an appropriate methodology for the incorporation of ES into HTA. As proposed by previous studies (25;48), the findings of this research indicate that the endeavor for an ES-HTA integration necessitates a multi-tiered, interdisciplinary strategy. This approach should encompass the participation of HTA practitioners at both national and institutional levels, decision-makers, environmental scientists, as well as clinical and biomedical engineers.

## Supporting information

Bobini and Cicchetti supplementary material Bobini and Cicchetti supplementary material

## Supplementary material

The supplementary material for this article can be found at http://doi.org/10.1017/S0266462324000631.
