# Supplementary material for: Integrating environmental sustainability into health technology assessment: an international survey of HTA stakeholders
Source: Int J Technol Assess Health Care. 2024 Nov 29;40(1):e64. doi: 10.1017/S0266462324000631 (PMC11703626; doi:10.1017/S0266462324000631)
Supplement: Bobini and Cicchetti supplementary material [file S0266462324000631sup001.docx]

**Survey**

PART I – HTA organizations’ progression towards an ES-HTA integration

1. Please write the name of your HTA agency. OPEN
2. Please describe your role inside your HTA agency. OPEN
3. Please write your email contact for further question: OPEN
4. Are you in favor about incorporating environmental sustainability into HTA? YES/NO
5. Is your agency somehow considering to include environmental sustainability dimension into HTA? YES/NO
6. Were you instructed by the national government to include the environmental sustainability dimension into HTA?
7. Why your agency is considering to include environmental sustainability dimension into HTA?
   1. Because environmental changes could directly affect people’s health.
   2. Because policy decision makers have broad mandates and objectives extending beyond health care.
   3. All of the above.
8. Has the integration of an environmental sustainability dimension into HTA been formally included in your agency's strategic objectives? YES/NO
9. Has your agency worked (or it is currently working) on integrating environmental sustainability/impacts dimension into HTA? YES/NO
10. Please describe how (method/approach) your agency is incorporating environmental sustainability/impacts into HTA. OPEN ANSWER
11. In order to integrate the environmental sustainability dimension into the HTA, what actions have been taken at organisational level?
    1. creation of a dedicated team by new recruitments
    2. creation of a dedicated team by reorganization of existing units
    3. assignment of the task to an existing team
    4. assignment of the task to an existing team and strengthening the team through new hires
12. What kind of backgrounds have those involved in the integration of environmental sustainability dimension into HTA?
    1. Economic
    2. Engineering
    3. Law
    4. Medicine
    5. Pharmacy
    6. Chemistry
    7. Other (please specify which one)

PART II – Possible trajectories in order to integrate environmental sustainability

1. Life Cycle Assessment (LCA) appears to be the prevailing approach in the scholarly discourse concerning the integration of ES into HTA. LCA offers a comprehensive perspective by evaluating environmental impacts throughout all stages of the life cycle, including raw material extraction, manufacturing, distribution, utilization, and eventual recycling or disposal. Moreover, two methods for conducting LCA have emerged: Environmentally Extended Input-Output Analysis (EEIOA), which utilizes country-specific economic input/output tables to estimate average carbon emissions associated with goods or services exchanged within a particular sector, and Process-based Life Cycle Assessment (P-LCA), which estimates carbon emissions in detail for each activity carried out within interconnected processes throughout the value chain.

**Please express your likelihood to adopt each approach in a scale where 1= very unlikely and 10= very likely.**


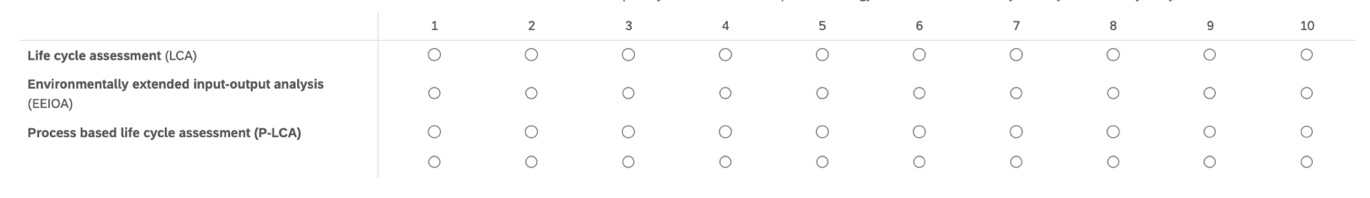


1. Assessing the environmental impact of health technologies requires the identification of outcome measures that quantify the environmental impact. At least three alternative methods emerge from the literature: a) Cost-utility analysis (CUA), which estimates the marginal health gains associated with the marginal environmental improvements of using one technology rather than another; b) Cost- benefit analysis (CBA), which converts all outcomes into monetary units, thereby capturing and allowing direct comparison between a wide range of social costs and benefits; c) Multicriteria decision analysis (MCDA), which encompasses a range of different methods, including, among others, the analytic hierarchy process, the analytic network process, the multi-attribute utility theory, the multi-attribute value theory, outranking, the social multicriteria evaluation, and the Technique For Order Of Preference By Similarity To Ideal Solution.

**Please express your likelihood to adopt each evaluation method in a scale where 1= very unlikely and 10= very likely.**


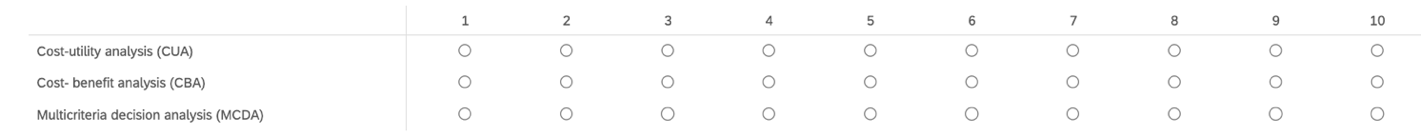


1. The following are some hindering factors for the developing the methods and processes to address the environmental impact question in an HTA. Please evaluate the importance of each factor according to a scale where 1=not important at all ad 10= very important.
   1. Lack of awareness about the relevance of including environmental sustainability into HTA
   2. Scarce availability of data or difficulty in tracing data back to a specific technology
   3. Environmental data collection can be labor intensive and time-consuming
   4. Absence of scientific consensus on the most appropriate integrative approach to capture environmental impacts of a technology
   5. Unfamiliarity with the approaches available
2. In accordance with HTAglossary.net, below are the possible types of technologies that can be evaluated through HTA. Please construct a ranking according to the priority of considering the environmental sustainability dimension in each of these application areas, where 1= item with which it is a higher priority to include the environmental sustainability dimension in assessment scope and 7= item with which it is a lower priority.
   1. Device
   2. Medicine
   3. Vaccine
   4. Procedure
   5. Program
   6. System
3. HTA models are often structured according to different domains which are then articulated into topics. Take for example the EUnetHTA’s HTA Core model, in which case the environmental sustainability dimension is only hinted at in two topics of the security domain: C0040 - what kind of risks for public and environment may occur when using the technology? C0064 – How can one reduce safety risks for environment?

Considering that, how do you think it would be more appropriate to integrate environmental sustainability into HTA?

- 1. To develop a separate domain dedicated to environmental sustainability.
  2. To integrate environmental sustainability transversally to the different domains by creating topics.

1. Please use this space for additional comments. OPEN ANSWER
